# Virulence Genes of *S. aureus* from Dairy Cow Mastitis and Contagiousness Risk

**DOI:** 10.3390/toxins9060195

**Published:** 2017-06-21

**Authors:** Giada Magro, Stefano Biffani, Giulietta Minozzi, Ralf Ehricht, Stefan Monecke, Mario Luini, Renata Piccinini

**Affiliations:** 1 Department of Veterinary Medicine, University of Milan, Via Celoria 10, 20133 Milan, Italy; giada.magro@unimi.it (G.Ma.); giulietta.minozzi@unimi.it (G.Mi.); 2 Italian Breeders Association (A.I.A.), via Tomassetti 9, 00161 Rome, Italy; ste.bif68@gmail.com; 3 Alere Technologies GmbH, Löbstedter Str. 103-105, 07749 Jena, Germany; ralf@clondiag.com (R.E.); stefan.monecke@clondiag.com(S.M.); 4 InfectoGnostics Research Campus, Philosophenweg 7, 07743 Jena, Germany; 5 Institute for Medical Microbiology and Hygiene (IMMH), Technische Universität Dresden, Fetscherstrasse 74, D-01307 Dresden, Germany; 6 Istituto Zooprofilattico Sperimentale della Lombardia e dell’Emilia Romagna-IZSLER, via Einstein, 26900 Lodi, Italy; mariovittorio.luini@izsler.it

**Keywords:** mastitis, dairy cow, *S. aureus*, virulence genes, microarray, prevalence

## Abstract

*Staphylococcus aureus* (*S. aureus*) is a major agent of dairy cow intramammary infections: the different prevalences of mastitis reported might be related to a combination of *S. aureus* virulence factors beyond host factors. The present study considered 169 isolates from different Italian dairy herds that were classified into four groups based on the prevalence of *S. aureus* infection at the first testing: low prevalence (LP), medium–low (MLP), medium–high (MHP) and high (HP). We aimed to correlate the presence of virulence genes with the prevalence of intramammary infections in order to develop new strategies for the control of *S. aureus* mastitis. Microarray data were statistically evaluated using binary logistic regression and correspondence analysis to screen the risk factors and the relationship between prevalence group and gene. The analysis showed: (1) 24 genes at significant risk of being detected in all the herds with infection prevalence >5%, including genes belonging to microbial surface components recognizing adhesive matrix molecules (MSCRAMMs), immune evasion and serine proteases; and (2) a significant correlation coefficient between the genes interacting with the host immune response and HP isolates against LP ones. These results support the hypothesis that virulence factors, in addition to cow management, could be related to strain contagiousness, offering new insights into vaccine development.

## 1. Introduction

*Staphylococcus aureus* (*S. aureus*) is recognized as one of the most relevant pathogens affecting dairy cattle herds. This contagious pathogen causes severe economic losses due to both clinical and subclinical mastitis [1]. The differences observed in strain contagiousness and the outcome of mastitis might be related to the absence, presence and combination of *S. aureus* virulence factors. Previous studies on the *S. aureus* genome showed a chromosomal co-linearity between the strains, with some genes harbored by all strains and others characterized by variable presence [2]. The bacterial genome comprises core and accessory genes (the latter being auxiliary and/or foreign genes that might be present in a given isolate, or not). In *S. aureus*, approximately 75% correspond to the core and 25% to the accessory genome. The core genes are usually associated with metabolism and other housekeeping functions common to all *S. aureus* strains. They also include variable genes not essential for growth and survival, but always present and characterized by lineage-specific gene sequences, such as some adhesion factors, surface binding proteins, exoenzymes and the capsule biosynthetic cluster. The accessory genome is the most variable genes class, consisting of genes that might have been introduced by horizontal gene transfer; among them are pathogenicity islands, phages, plasmids, transposons and chromosomal cassettes [2]. In particular, the staphylococcal chromosomal cassettes carry methicillin, fusidic acid or heavy metal resistances, and recombinase genes, which facilitate the horizontal gene transfer across the genus *Staphylococcus* [3]. The severity of *S. aureus* infection often depends on the variable genes. Haemolysin beta (*hlb*) increases the adherence of *S. aureus* to bovine mammary epithelial cells and cytotoxicity [4], while different enzymes (such as hyaluronidase, proteases and nucleases), non-enzymatic activators (such as coagulase or staphylokinase) or exotoxins (such as cytolytic toxins, exfoliative toxins, leukocidins, enterotoxins, enterotoxin-like proteins and toxic shock syndrome toxin-1) promote the bacterial escape from host immune response. The combination of these factors seems to be crucial to the outcome of mastitis [5]. In the last years, most of these virulence factors have been identified and their presence investigated in dairy cow isolates. Staphylococcal enterotoxins (SE) act as superantigens, stimulating T-lymphocytes and the release of large amounts of cytokines that can cause severe inflammation, but their role in the intramammary infections is still unclear. Indeed, previous studies showed variable frequencies of SE genes in bovine mastitis isolates from different countries [6,7,8]. Among *S. aureus* exotoxins, the bicomponent leukocidins are pore-forming molecules targeting bovine PMNs. Different leukocidin variants have been demonstrated in strains of bovine origin, such as *lukS*/*lukF* (γ-hemolysin), *lukD*/*lukE*, and especially *lukM*/*lukF–PV(P83*) [9,10,11]. The large array of *S. aureus* virulence factors also includes the production of microbial surface components recognizing adhesive matrix molecule (MSCRAMM) proteins, which adhere to the extracellular matrix [12]. Among them, some genes are involved in biofilm formation, such as clumping factor A and B, fibrinogen-binding protein, fibronectin-binding protein A and B [13], while the *S. aureus* surface protein G is implicated in intercellular auto-aggregation [14], as well as the serine–aspartate repeat proteins, which belong to a cluster of cell wall-anchored proteins important for *S. aureus* [15]. After the adhesion, proteases seem to be crucial, because they can cleave host proteins and allow staphylococcal transition from adhesive to invasive phenotype [16]. A deep knowledge of the entire pattern of virulence factors and its variability in bovine *S. aureus* isolates is still lacking. Also, the correlation between strain virulence, meaning the presence of virulence factors, and intramammary infection prevalence at herd level is poorly understood. In the present study, we characterized *S. aureus* strains collected in Italian dairy herds, using DNA-microarrays analysis, and investigated the association among virulence factors and strain prevalence at herd level. The final goal was to identify the genes most involved in a high prevalence of intramammary infections, in order to develop new strategies for the control of *S. aureus* mastitis, among them the possible identification of new vaccine targets.

## 2. Results

Out of 169 *S. aureus* strains tested, 157 (92.9%) were MSSA and 12 (7.1%) were MRSA. The isolates were distributed in the four classes of prevalence of *S. aureus* mastitis as follows: 45 (26.63%) were in the Low Prevalence (LP) herds, 44 (26.03%) in Medium–Low Prevalence (MLP), 33 (19.53%) in Medium–High Prevalence (MHP) and 47 (27.81%) in High Prevalence (HP). The herds were similar in the extensive animal husbandry, while the average number of lactating cows was not homogeneous among prevalence classes. The main characteristics of the herds in the four groups are reported in Table 1.

The results of the microarray analysis performed on *S. aureus* isolates are shown in Table 1, while the diffusion of the different Clonal Complexes (CCs) throughout the classes of prevalence are summarized in Table 2. 

CC8 was the most frequently isolated group of *S. aureus* and mostly related with the three classes of medium and high prevalence, whereas CC398 was typical of LP herds. The other most commonly represented *S. aureus* groups were CC97 (12.4%) and ST126 (8.3%).

Twenty-six genes were detected in all the strains: among them, we found several important virulence factors, such as leukocidin/γ-haemolysin genes *lukF/S* and the homologue *lukX/Y* (=*lukA/B* or *lukG/H*)/*hlgA*; the genes encoding the proteases aureolysin (*aur*), glutamyl endopeptidase (*sspA*) and staphopain B (*sspB*); the genes encoding staphylococcal exotoxin-like proteins *setC* (*selX*) and *setB*; and the hyaluronate lyase genes (*hys*). MSCRAMMs such as clumping factor A (*clfA*) and B (*clfB*), the cell surface elastin-binding protein (*ebpS*), the enolase enzyme (*eno*), and the van Willebrand factor binding protein (*vwb*) were also detected in all isolates. On the other side, certain genes were never detected: most of them encoded antibiotic resistance, exfoliative toxins, or one of the capsule type 1 locus genes (*capK1*). Overall, 75% of the strains were assigned to *agr* group *I*, 20% to *agr II*, and the remaining 5% to *agr III*. Different clonal complexes carried *agr I* (CC101, CC133, CC20, CC398, CC522, CC8, CC97, ST71 and ST72) or *II* (CC479, CC5, CC705 and ST126), respectively. Only CC1 harbored *agr III*. The binary logistic regression analysis (BLR) performed on each gene separately, using LP as the reference class, identified the following genes as related to the herds with *S. aureus* infection prevalence >5%: three enterotoxins with the same plasmid origin (*sed*, *ser*, *sej*); a leukocidin (*lukD/E*); the disrupted β-haemolysin (*hlb*) and the genes inserted by the truncating phage, i.e., staphylokinase (*sak*) and the staphylococcal complement inhibitor (*scn*); proteases (*splA*, *splB* and *splE* and an allelic variant of aureolysin); and MSCRAMMs, such as the fibrinogen-binding protein (*fib*), elastin-binding protein (*ebpS*) and allelic variants of clumping factor B (*clfB*), fibronectin-binding protein A and B (*fnbA* and *fnbB*), *S. aureus* surface protein G (*sasG*), serine–aspartate repeat protein D (*sdrD*) and the van Willebrand factor-binding protein (*vwb*). These genes are listed in Table 3. 

**Figure 1:**
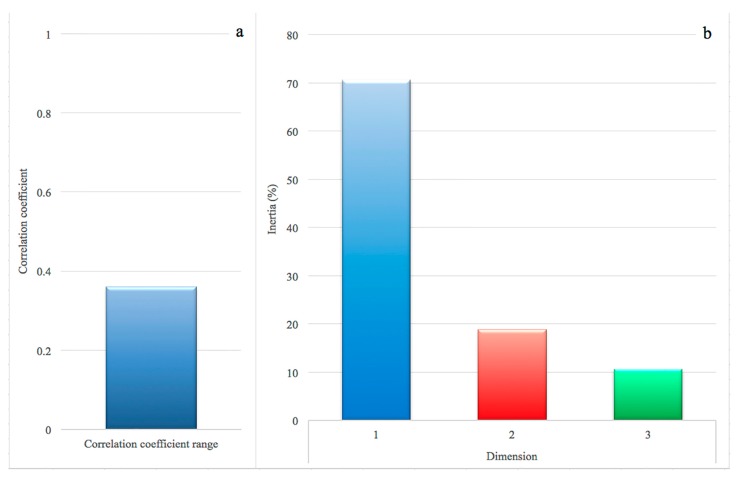
Correlation coefficient between 169 genes and four prevalence classes of intramammary infections by *S. aureus*. (**a**) correlation coefficient: a value above 0.20 suggests a moderate to strong correlation; (**b**) proportion of variances retained by the first three dimensions.

In the correspondence analysis (CA), the association between rows (genes) and columns (prevalence class) was 0.36, confirmed by a Pearson’s Chi-squared test (chi-square = 1612.4, df = 750, *p* < 0.0001). The results of the magnitude of correlation are shown in Figure 1a: the threshold of 0.20 is considered as an indicator of correlation [17,18]. Regarding the observed variability (*inertia*) and its decomposition in the dimensions, the first, second and third dimension explained 70.6%, 18.8% and 10.6% of the observed variance, respectively (Figure 1b). 

**Figure 2:**
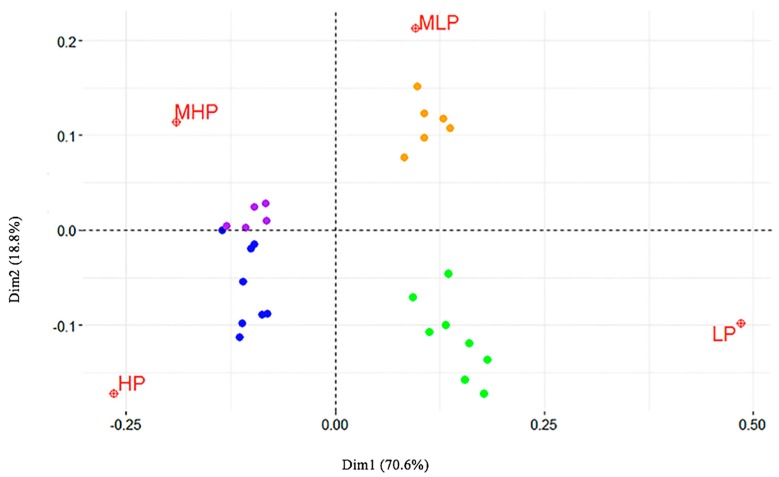
Relative position of the genes (points) in the four prevalence classes (space: low prevalence, LP; medium–low, MLP; medium–high, MHP; high prevalence, HP).

The Malinvaud’s test (1987) was applied to identify the optimal number of dimensions to retain, and showed that the first two dimensions were significant (*p* < 0 and *p* < 0.0001, respectively). Following Greenacre [19], these dimensions were then used to produce a *biplot* (Figure 2), displaying the relative position of the row points (i.e., the genes) in the space (i.e., the prevalence classes). The relative distance between points of different type is the *correspondence* between the categories that made up the table. The distance between each class and 0, such as between data points and 0, indicates the degree of similarity: HP and LP are much more distant from 0, when compared to MHP and MLP. For this reason, we considered only the two extreme classes. 

According to Greenacre [18], a rule of thumb to select the most important row variables that are related to each column variable is to use a threshold based on the average contribution, defined as $\frac{100}{number\;of\;rows}*10$. Applying this formula to our data set, the threshold was = 6, meaning that 10 genes were to be considered for LP, as well as for HP (Table 4). 

## 3. Discussion

The variability in the virulence of *S. aureus* strains plays a central role in the development of intramammary infections of the dairy cow and in the subsequent spread to other animals. In order to identify the genes that might be mostly implicated in the virulence of the strains, we used the microarray technology [20] to characterize, on the molecular level, 169 isolates from dairy cow mastitis, collected in 60 herds located in different Italian regions. The results were then associated with the prevalence of *S. aureus* intramammary infections at herd level and statistically analyzed using two different approaches, the BLR and the CA. The former detected and measured the strength of the patterns of association between each single gene and the LP class of prevalence; the latter investigated the pattern of relationships of several categorical dependent variables, showing which gene was dominant across each prevalence group and graphically representing these relative frequencies in a low-dimensional space. In comparison with previous studies [11,12,13,14,15,16,17,18,19,20,21], the present one considered a higher number of isolates and, especially, the ability of the strain to spread within the herd. We decided to use the array technology because it is the best method for cost-benefit relation: proper bioinformatics was performed with consensus probes for all targets and, even if such a method cannot differentiate between functional and non-functional genes, it is very difficult to elucidate this topic. Indeed, a gene might be active when the infection process starts, and later become obsolescent once the infection flourishes. On the other side, NGS technology also has many disadvantages, starting from the analysis time, to the loss of short repeats and the scarcity of standards. Regarding the expression of the factor, the presence of a gene is not always correlated with its expression; however, the absence always means lack of expression. Therefore, the analysis of presence/absence of genes coding for virulence factors in cows affected by *S. aureus* mastitis represents the first step, which could be possibly followed by functionality studies.

Regarding the distribution in CCs, a notable result was the finding of 8.3% ST126. This represents a cow-associated lineage that has been found in the Americas and in Southern Europe (see MLST database) [22], while studies from Central and Northern Europe failed to detect it [20,21,22,23]. Isolates of this lineage were observed in LP, but also in MHP and HP herds. 

Both MSSA and MRSA strains grouping in CC398 belonged to the LP class of prevalence. This is in accordance with another recent Italian study demonstrating that CC398-MRSA was associated with low prevalence infections in dairy herds [24]. Since MSSA as well as MRSA from this lineage are common in a variety of other livestock animals, especially in poultry and pigs, it might be assumed that these isolates represent spill-over from other farm animals, whereas humans as well as rodents or flies might have served as vectors. 

We found a high prevalence of CC8-MSSA in the herds considered in the present study. CC8-MSSA is a common strain in humans, but it was already reported as a strain frequently causing bovine mastitis in Western Switzerland, suggesting a recent host shift from humans to cows concurrent with a loss of the ability to colonize humans [25]. 

The distribution of the CCs in the classes of infection prevalence was not uniform, reflecting a higher risk of contagiousness for certain lineages in comparison to others. For this reason, the statistical association of some virulence factors with HP herds is of interest from a mastitis control perspective, despite the possible bias due to CC distribution. 

The CA clearly distinguished LP and HP as extreme prevalence classes. Interestingly, LP genes were mostly allelic variants only found in MRSA strains: this finding is in accordance with a recent paper [24] demonstrating that livestock MRSA are typically not diffusive. Regarding the carriage of *cna* by LP strains, the gene has been suggested not to play an important role in *S. aureus* intramammary infections [26]. Different genes were significantly related to the strains isolated in those herds, where the prevalence of *S. aureus* mastitis was above 5%. They were involved in the evasion of host immune response (*sak* and *scn*), in the killing of phagocytes (*lukD/E*) or displayed superantigenic activity (*sed*, *ser* and *sej*); some genes were involved in tissue adhesion (*fib*) and invasion (*splA*, *splB*, *splE*). This result was strengthened by the CA, which highlighted a significant correlation in the distribution of *sed*, *ser*, *sej*, *sak* and *scn* with prevalence of *S. aureus* intramammary infections exceeding 40%. *Sed*, *ser*, and *sej* belong to a cluster harbored by different CCs, out of them CC151 and CC479 were indicated as the most frequent ones [11]. The genes *sea*, *sak* and *scn*, which are carried by β-hemolysin-converting bacteriophages, were present uniquely in some CC8 strains; their prevalence was higher than in other studies on bovine isolates, although lower than in studies on isolates from humans [20,21,22,23,24,25,26,27]. Accordingly, a recent paper reported that CC8 strains of bovine or human origin differed for the mobile genetic elements, among them the β-hemolysin-converting prophages: all bovine-only isolates were devoid of such prophages [28], probably because the untruncated *hlb* is necessary in ungulates for the different structure of erythrocyte membranes. This also supports the concept of a recent transmission from humans into cows [25]. Nevertheless, a clear contagious trait of CC8 strains has yet to be identified. 

The role of enterotoxins in bovine mastitis is not completely elucidated, but they are supposed to promote the efficacy of *S. aureus* infections in cattle. Leukocidins target PMNs, weakening the host immune response. Both variants *lukD*/*lukE* and *lukF-PV(P83)/lukM* have been associated with bovine mastitis [10,11]. Accordingly, one or both were detected in most CCs, while they were absent in CC398. Even though *lukF-PV(P83)/lukM* was suggested to play an essential role in the etiology of bovine mastitis, our results showed a higher frequency of *lukD/E* and a significant correlation with the risk of being detected in MP and HP. This result could possibly be explained by an over-expression of this leucocidin variant, in the absence of *lukF-PV(P83)/lukM*. However, it also could be interpreted as accidental circumstance related to an ongoing epidemic of *lukD/E*-positive, *lukF-PV(P83)/lukM*-negative CC8 clone, assuming that its current spread was linked to factors other than leukocidin activity. 

Altogether, the genetic array demonstrated in the HP *S. aureus* strains could counteract the efficacy of mammary immune response, enabling the microorganism to promptly infect the herd. The MSCRAMM family includes different adhesins, which are essential for initial stages of infection. Among them, the fibrinogen-binding protein (*fib*) demonstrated a high risk of being detected in herds with *S. aureus* prevalence >5%, suggesting an involvement in strain contagiousness. The result differs from what was reported in a previous study, which described the gene as not significantly associated with mastitis [6]. Other adhesins such as *clfA* and *clfB*, *epbS* and *vwb* were harbored by all strains, contrarily to previous studies, reporting variable frequencies [27,28,29,30]. Interestingly, an allelic variant of *clfB*, *epbS* and *vwb* was prevalence-related, as it was detected in more contagious strains. The significantly different distributions of MSCRAMM allelic variants in the four groups of *S. aureus* prevalence and the correlation with HP herds for some of them might be the result of selective pressure. Indeed, different environments and management practices could amplify differences between strain virulence patterns [21]. A *sdrD* allelic variant was also included in the group of the genes at risk and correlated with HP; however, the role of this protein in bovine mastitis is still unclear, even though a significant association between *sdrD* and clinical mastitis was demonstrated [30]. Proteases promote invasion through degrading some of the cell surface components, such as fibronectin, fibrinogen and elastin [31]. The literature regarding the role of proteases in dairy cow mastitis is scarce: one study highlighted the high frequency of *splA* and *sspA*, but the variability of *splE* [30]. In human medicine, an association between the presence of *splA*/*splB* and *S. aureus* invasive endocarditis was found in hospitalized patients [32]. Our data seem to support those results, since a significant difference in the frequency of *spl*s was detected among the groups of mastitis prevalence, with a higher risk of carriage by more contagious strains. 

The control of *S. aureus* mastitis is mostly based on the separation of infected cows; since two vaccines are available on the market, and the level of protection offered is not overall the same, because of the important role played by herd factors [33]. Considering the results of the present study, strain contagiousness appears to be related to an entire pattern of virulence factors, which target both adhesion/invasion of mammary tissue and the immune response of the gland. Such results offer new insights in the development of an innovative vaccine against *S. aureus* mastitis. Nevertheless, it should be highlighted that a multi-centre study across several countries is recommended in order to find a “least common denominator” for the genetic outfit of *S. aureus* causing bovine mastitis in different countries.

## 4. Materials and Methods

### Herds, Sampling and Microarray Analysis

The study considered 169 *S. aureus* isolates, collected in 60 dairy herds located in different Italian regions between 2006 and 2014. All cows were intensively reared in free stalls with cubicles; only in two herds were the animals housed in stanchion barns. Following the intensive husbandry, the medium age of the cows was overall similar, around 4.2–5 years. The lactating cows ranged from 15–245 and were undergoing a control program for *S. aureus* mastitis. Quarter milk samples were aseptically taken from all lactating cows and delivered to the laboratory. Most cows did not show signs of clinical mastitis. Somatic cells (SCC) were counted on a Bentley Somacount 150 (Bentley Instruments, Chaska, MN, USA), and bacteriological analysis was performed [34]. Coagulase-positive staphylococcal strains were confirmed as *S. aureus* using a duplex real-time PCR assay [35] and then frozen at −80 °C in a Microbank bacterial preservation system (Thermo Fisher Scientific Inc, Waltham, MA, USA). The prevalence of *S. aureus* infections at herd level was calculated and 1 to 4 isolates per herd were included in the study, depending on the prevalence and on colony morphology on blood agar plate.

Bacterial DNA was extracted using DNeasy kit (QIAgen, Hilden, Germany), with the addition of lysostaphin (5 mg/mL; Sigma-Aldrich, St. Luis, MO, USA) for bacterial lysis. Amount and quality of DNA samples were measured on a NanoDrop ND-1000 spectrophotometer (Nano-Drop Technologies, Wilmington, DE, USA). 

A DNA microarray (*S. aureus* Genotyping Kit 2.0; Alere Technologies GmbH, Jena, Germany) was used to genetically characterize the *S. aureus* strains. The tool detects a total of 330 different sequences, including accessory gene regulator alleles, genes coding for virulence factors and for microbial surface components recognizing adhesive matrix molecules (MSCRAMMs), capsule type-specific genes, and numerous antimicrobial resistance genes. Probes for the methicillin-resistance genes *mecA* and *mecC* are also included. The overall pattern was analyzed automatically for the presence or absence of specific genes and compared to a database of strain profiles allowing the assignment to Clonal Complexes (CC). The genotyping service was performed at Alere Technologies (Jena, Germany). 

## 5. Statistical Analysis

Four classes were defined a priori, based on the prevalence of intramammary infections by *S. aureus* found in the first sampling of all lactating cows in each herd: low prevalence (LP) when <5% cows tested positive, medium–low (MLP) or medium–high (MHP) when the infection ranged 5.1–24% or 24.1–40%, respectively, and high prevalence (HP) when >40% cows had at least one quarter infected by *S. aureus*. Prior to the statistical analyses, the genes that did not show any variation (i.e., only positive or negative results) were excluded, eventually retaining 169 genes.

### 5.1. Binary Logistic Regression (BLR) and Risk Factors Calculation

Binary logistic regression analysis was conducted with the SPSS software (SPSS Inc., Chicago, IL, USA). Each gene was analyzed both separately and independently. The dependent variable (dichotomous) was the presence or absence of the specific gene, while the prevalence classes were treated as categorical and corrected for the number of lactating cows per herd. The LP class was used as reference for the analysis; *p*-values lower than 0.05 were considered significant. Frequencies of genes within each class of prevalence were also estimated with the SPSS software as shown in Table 2. For each variable, the regression coefficient (*B*) and the Wald test result (used to test significance) are shown. Further Odds ratio (for each variable category) has been estimated within the SPSS software environment. 

### 5.2. Data Editing and Correspondence Analysis (CA)

contingency table was created counting the number of genes (row) per each prevalence herd class (column). The final contingency table is represented by the I×J matrix X, whose generic element $x_{i,j}$ gives the number of observations that belong to the ***i*_th_** level of the first nominal variables (169 genes) and the ***j***th level of the second nominal variable (four herd prevalence classes). The grand total of the table is noted ***N***. The goal of CA is to transform this contingency table into two sets of factor scores (one for the rows and one for the columns), which give the best representation of the similarity structure of the rows and the columns of the table. In order to calculate the factor scores, the contingency table is first transformed into a probability matrix ***Z***, computed as $Z=N^{-1}X$. Then, following Abdi Béra [36] and Nenadić and Greenagre [37], the factor scores are obtained from the following *generalized* singular value decomposition (**GSVD**):

$$\left(Z-rc^{T}\right)=P∆Q^{T}\;with\;P^{T}D_{r}^{-1}P=Q^{T}D_{c}^{-1}Q=I$$

where ***r*** and ***c*** denote the vectors of the row and column totals of ***Z***, respectively. The subtraction of $rc^{T}$ from Z centers the matrix while P, ∆ e Q are the left and right singular vectors, and the diagonal matrix of singular values, respectively. From the **GSVD** the factor scores are obtained as:

$$F=D_{r}^{-1}P∆\;and\;G=D_{c}^{-1}Q∆$$

An important statistic in CA is the total variance of the data matrix or *inertia* [18], which is calculated on relative observed and expected frequencies:

$$Inertia=\emptyset^{2}=\sum_{i=1}^{I}\sum_{j=1}^{J}\frac{{\left(p_{ij}+r_{i}c_{j}\right)}^{2}}{r_{i}c_{j}}$$

CA was implemented using the *ca* [37], *FactorMineR* [38], *vcd* [39] and *CAinterprTools* [40] libraries of R [41].

## 6. Conclusions

The current study was performed on a representative database of Italian isolates and showed that the herd prevalence of intramammary infections caused by *S. aureus* could be linked to specific combinations of virulence genes, in addition to management practices. The most important findings are: (1) some genes were always absent, or evenly distributed in all strains considered; (2) genes belonging to MSCRAMMs (*fib*, *fnbA*, *fnbB*, *sdrD* and *sasG*) and the serine proteases had an increased risk of being detected in more contagious strains; (3) a heterogeneous group of genes interacting with the host immune response, including *sed*, *ser*, *sej*, *sak* and *scn* was correlated with the herds characterized by the highest prevalence of *S. aureus* mastitis. In conclusion, the results of the present study highlighted that a specific pattern of genes could be responsible for the higher contagiousness of the strains. Such findings can therefore contribute to the development of a new vaccine for dairy cow mastitis by *S. aureus*. 

## Figures and Tables

**Table 1:** Main characteristics of the herds in the four prevalence classes of *S. aureus* intramammary infections. LP, low prevalence herds; MLP, medium-low prevalence; MHP, medium-high; HP, high prevalence.

Prevalence Class	Cubicle Houses (No.)	Stanchion Barns (No.)	Lactating Cows, Average (min.–max.)
LP	20	-	96.2 (15–245)
MLP	15	1	67.6 (40–130)
MHP	10	1	52.0 (15–120)
HP	13	-	70.6 (15–195)

**Table 2:** Overall distribution of the different Clonal Complexes (CCs) of *S. aureus* and CC distribution in the classes of prevalence of mastitis by *S. aureus* at herd level (low prevalence, LP; medium–low, MLP; medium–high, MHP; high prevalence, HP).

CC	Number of Strains	Overall Distribution of CCs (%)	Distribution of CCs in the Groups of Prevalence (%)			
			LP	MLP	MHP	HP
CC1	7	4.14	4.55	2.22	0	8.51
CC5	4	2.37	0	0	12.12	0
CC8	70	**41.42**	11.36	**46.67**	**42.42**	**63.83**
CC20	4	2.37	6.82	0	3.03	0
CC97	21	12.43	9.09	22.22	6.06	10.64
CC101	1	0.59	0	0	0	2.13
CC133	3	1.77	6.82	0	0	0
CC398	24	14.20	**36.36**	8.89	0	8.51
CC479	5	2.96	6.82	4.44	0	0
CC522	2	1.18	0	4.44	0	0
CC705	12	7.10	9.09	11.11	9.09	0
ST126	14	8.28	4.55	0	27.27	6.38
ST72	1	0.59	2.27	0	0	0
*agr IV*, undef. CC	1	0.59	2.27	0	0	0

**Table 3:** Relative risk of detection of the genes with a significant different distribution in the four classes of prevalence of intramammary infections by *S. aureus*, using the lower frequency class as reference. MLP, herds with prevalence 5.1–24%; MHP, prevalence 24.1–40%; HP, prevalence >40.1%.

Genes	Relative Risk to the LP Class				
	Sign.	MLP	MHP	HP	
*sea*	0.034	1.61	3.41	5.53	enterotoxin A
*sed*	0.001	1.91	4.84	7.97	enterotoxin D
*ser*	0.001	1.64	4.92	8.05	enterotoxin R
*sej*	0.001	1.64	4.92	8.05	enterotoxin J
*lukD*	0.001	5.53	16.12	6.76	leukocidin D component
*lukE*	0.010	3.50	>25.00	6.76	leukocidin E component
*hlb* probe 3	0.007	4.66	4.126	6.09	haemolysin beta
*sak*	0.020	1.50	3.69	5.78	staphylokinase
*scn*	0.020	1.50	3.69	5.78	staphylococcal complement inhibitor
*splA*	0.036	3.65	>25.00	5.71	serin–protease A
*splB*	0.050	3.56	>25.00	4.90	serin–protease B
*splE*	0.000	5.04	7.25	10.47	serin–protease E
*aur* ^★^	0.050	3.56	>25.00	4.90	aureolysin
*fib*	0.050	3.56	>25.00	4.90	fibrinogen-binding protein
*ebpS* probe 612	0.004	5.48	>25.00	6.75	cell surface elastin-binding protein
*clfB* ^▲^	0.000	8.60	11.74	17.40	clumping factor B
*fnbA* ^✦^	0.000	2.94	7.11	7.21	fibronectin-binding protein A
*fnbB* ^✦^	0.000	16.99	13.93	31.81	fibronectin-binding protein B
*sasG* ^▲^	0.000	5.82	3.69	6.19	*S. aureus* surface protein G
*sasG* *	0.000	5.26	2.23	6.54	
*sdrC* ^✦^	0.002	3.97	2.29	5.68	Serine–aspartate repeat protein C
*sdrC* *	0.000	3.28	12.75	15.22	
*sdrD* ^※^	0.002	4.27	3.25	7.52	Serine–aspartate repeat protein D
*vwb* ^※^	0.000	4.55	3.38	12.78	van Willebrand factor-binding protein

* Indicates other allelic variants than MRSA252 (CC30). ^▲^ indicates the allelic variant shared by COL (CC8) and Mu50 (CC5). ^✦^ indicates the allelic variant of COL (CC8). ^★^ indicates other allelic variants than MRSA252 (CC30) and RF122 (CC151/CC705). ^※^ indicates the allelic variant shared by COL (CC8) and MW2 (CC1).

**Table 4:** Genes significantly correlated with the extreme classes of intramammary infection prevalence (low prevalence, LP; high prevalence, HP).

Gene	Prevalence Class	
*ebpS* ^♦^	LP	cell surface elastin-binding protein
*tetM*	LP	tetracycline resistance
*aur* ^Y^	LP	aureolysin
*fib* ^Y^	LP	fibrinogen-binding protein
*vga* ^†^	LP	ATP-binding protein, streptogramin A resistance
*cna*	LP	collagen-binding adhesin
*dfrS1*	LP	dihydrofolate reductase type 1
*clfB* ^◈^	LP	clumping factor B
*capJ1*	LP	O antigen polymerase
*fexA*	LP	chloramphenicol/florfenicol exporter
*fnbB* ^♦^	HP	fibronectin-binding protein B
*sej*	HP	enterotoxin J
*ser*	HP	enterotoxin R
*sed*	HP	enterotoxin D
*vwb* ^※^	HP	van Willebrand factor-binding protein
*sdrD* ^※^	HP	serine-aspartate repeat protein D
*fnbA* ^♦^	HP	fibronectin-binding protein A
*sak*	HP	staphylokinase
*scn*	HP	staphylococcal complement inhibitor
*sea*	HP	enterotoxin A

^♦^ indicates the allelic variant of Sequence Type 45. ^Y^ indicates the allelic variant of MRSA252 (CC30). ^†^ indicates the allelic variant of BM3327. ^◈^ indicates the allelic variant of MW2 (CC1). ^※^ indicates the allelic variant shared by COL (CC8) and MW2 (CC1). ^♦^ indicates the allelic variant of COL (CC8).

## Supplementary Materials

The following are available online at www.mdpi.com/2072-6651/9/6/195/s1, Table S1.: Complete results of the microarray analysis performed on *S. aureus* isolates from 60 dairy herds in Italy.
