# Supplementary material for: Virulence Genes of S. aureus from Dairy Cow Mastitis and Contagiousness Risk
Source: Toxins (Basel). 2017 Jun 21;9(6):195. doi: 10.3390/toxins9060195 (PMC5488045; doi:10.3390/toxins9060195)
Supplement: Supplementary file 1 [file toxins-09-00195-s001.pdf]

# Supplementary Materials: Virulence Genes of S. aureus from Dairy Cow Mastitis and Contagiousness Risk

**Giada Magro, Stefano Biffani, Giulietta Minozzi, Ralf Ehricht, Stefan Monecke, Mario Luini and Renata Piccinini**

**Table S1.** Complete results of the microarray analysis performed on *S. aureus* isolates from 60 dairy herds in Italy.

| Genes | | Classes of prevalence (Frequency %) LP | MLP | MHP | HP | |
|---|---|---|---|---|---|---|
| sarA | POS | 1,00 | 1,00 | 1,00 | 1,00 | staphylococcal accessory regulator A |
| saeS | POS | 1,00 | 1,00 | 1,00 | 1,00 | histidine protein kinase, sae locus |
| vraS | POS | 1,00 | 1,00 | 1,00 | 1,00 | sensor protein |
| agrB-I | neg | 0,25 | 0,20 | 0,49 | 0,15 | accessory gene regulator allele I |
| | POS | 0,75 | 0,80 | 0,52 | 0,85 | |
| agrC-I | neg | 0,25 | 0,18 | 0,49 | 0,15 | |
| | POS | 0,73 | 0,82 | 0,52 | 0,85 | |
| agrD-I | neg | 0,27 | 0,18 | 0,49 | 0,15 | |
| | POS | 0,73 | 0,82 | 0,52 | 0,85 | |
| agrB-II | neg | 0,80 | 0,84 | 0,52 | 0,94 | accessory gene regulator allele II |
| | POS | 0,21 | 0,16 | 0,49 | 0,06 | |
| agrC-II | neg | 0,80 | 0,84 | 0,52 | 0,94 | |
| | POS | 0,21 | 0,16 | 0,49 | 0,06 | |
| agrD-II | neg | 0,80 | 0,84 | 0,52 | 0,94 | |
| | POS | 0,21 | 0,16 | 0,49 | 0,06 | |
| agrB-III | neg | 0,89 | 0,98 | 1,00 | 0,92 | accessory gene regulator allele III |
| | POS | 0,07 | 0,02 | 0,00 | 0,09 | |
| agrC-III | neg | 0,96 | 0,98 | 1,00 | 0,92 | |
| | POS | 0,05 | 0,02 | 0,00 | 0,09 | |
| agrD-III | neg | 0,96 | 0,98 | 1,00 | 0,92 | |
| | POS | 0,05 | 0,02 | 0,00 | 0,09 | |
| agrB-IV | neg | 0,77 | 0,56 | 0,64 | 0,38 | accessory gene regulator allele IV |
| | POS | 0,23 | 0,44 | 0,36 | 0,62 | |
| agrC-IV | neg | 0,98 | 1,00 | 1,00 | 1,00 | |
| | POS | 0,02 | 0,00 | 0,00 | 0,00 | |
| hld | POS | 1,00 | 1,00 | 1,00 | 1,00 | haemolysin delta |
| blaZ | neg | 0,46 | 0,44 | 0,33 | 0,32 | beta-lactamase |
| | POS | 0,55 | 0,56 | 0,67 | 0,68 | |
| blaI | neg | 0,46 | 0,44 | 0,33 | 0,32 | beta lactamase repressor (inhibitor) |
| | POS | 0,55 | 0,56 | 0,67 | 0,68 | |
| blaR | neg | 0,46 | 0,44 | 0,33 | 0,32 | beta-lactamase regulatory protein |
| | POS | 0,55 | 0,56 | 0,67 | 0,68 | |
| erm(A) | neg | 1,00 | 0,98 | 1,00 | 1,00 | rRNA adenine N-6-methyltransferase, erythromycin/clindamycin resistance |
| | POS | 0,00 | 0,02 | 0,00 | 0,00 | |
| erm(B) | neg | 0,98 | 1,00 | 1,00 | 1,00 | erythromycin/clindamycin resistance |
| | POS | 0,02 | 0,00 | 0,00 | 0,00 | |
| erm(C) | neg | 1,00 | 1,00 | 1,00 | 0,98 | erythromycin/clindamycin resistance |
| | POS | 0,00 | 0,00 | 0,00 | 0,02 | |

| | | | | | | |
|---|---|---|---|---|---|---|
| lnu(A) | neg | 1,00 | 1,00 | 1,00 | 1,00 | lincosamid-nucleotidyltransferase |
| msr(A) | neg | 1,00 | 1,00 | 1,00 | 1,00 | energy-dependent efflux of erythromycin |
| mef(A) | neg | 1,00 | 1,00 | 1,00 | 1,00 | macrolide efflux protein A |
| mph(C) | neg | 1,00 | 1,00 | 1,00 | 1,00 | probable lysylphosphatidylglycerol synthetase |
| vat(A) | neg | 1,00 | 1,00 | 1,00 | 1,00 | virginiamycin A acetyltransferase |
| vat(B) | neg | 1,00 | 1,00 | 1,00 | 1,00 | acetyltransferase inactivating streptogramin A |
| vga(A) | neg | 0,93 | 0,98 | 0,97 | 1,00 | ATP-binding protein, streptogramin A resistance |
| | POS | 0,07 | 0,02 | 0,03 | 0,00 | |
| vga(A) (BM 3327) | neg | 0,66 | 0,96 | 1,00 | 0,92 | |
| | POS | 0,34 | 0,04 | 0,00 | 0,09 | |
| vgB(A) | neg | 1,00 | 1,00 | 1,00 | 1,00 | virginiamycin B hydrolase |
| aacA-aphD | neg | 1,00 | 1,00 | 1,00 | 1,00 | bifunctional enzyme Aac/Aph, gentamicin resistance |
| aadD | neg | 0,96 | 1,00 | 1,00 | 1,00 | aminoglycoside adenyltransferase, tobramycin resistance |
| | POS | 0,05 | 0,00 | 0,00 | 0,00 | |
| aphA3 | neg | 1,00 | 0,98 | 1,00 | 0,98 | 3'5'-aminoglycoside phosphotransferase, neo-/kanamycin resistance |
| | POS | 0,00 | 0,02 | 0,00 | 0,02 | |
| sat | neg | 1,00 | 0,98 | 1,00 | 0,98 | streptothricine acetyltransferase |
| | POS | 0,00 | 0,02 | 0,00 | 0,02 | |
| dfrS1 | neg | 0,86 | 1,00 | 1,00 | 1,00 | dihydrofolate reductase type 1 |
| | POS | 0,14 | 0,00 | 0,00 | 0,00 | |
| far1 | neg | 1,00 | 1,00 | 1,00 | 1,00 | fusidic acid resistance |
| fusC (Q6GD50) | neg | 1,00 | 1,00 | 1,00 | 1,00 | hypothetical protein associated with fusidic acid resistance |
| mupA | neg | 1,00 | 1,00 | 1,00 | 1,00 | mupirocin resistence protein |
| tet(K) | neg | 1,00 | 0,96 | 0,94 | 1,00 | tetracycline resistance |
| | POS | 0,00 | 0,04 | 0,06 | 0,00 | |
| tet(M) | neg | 0,59 | 0,96 | 1,00 | 0,92 | |
| | POS | 0,41 | 0,04 | 0,00 | 0,09 | |
| cat (total) | neg | 0,96 | 1,00 | 1,00 | 1,00 | chloramphenicol acetyltransferase |
| | POS | 0,05 | 0,00 | 0,00 | 0,00 | |
| cat (pC221) | neg | 0,96 | 1,00 | 1,00 | 1,00 | |
| | POS | 0,05 | 0,00 | 0,00 | 0,00 | |
| cat (pc223) | neg | 1,00 | 1,00 | 1,00 | 1,00 | |
| cat (pMC524/pC194 ) | neg | 1,00 | 1,00 | 1,00 | 1,00 | |
| cat (pSBK203R) | neg | 1,00 | 1,00 | 1,00 | 1,00 | |
| cfr | neg | 1,00 | 1,00 | 1,00 | 1,00 | 23S rRNA methyltransferase |
| fexA | neg | 0,93 | 1,00 | 1,00 | 1,00 | chloramphenicol/florfenicol exporter |
| | POS | 0,07 | 0,00 | 0,00 | 0,00 | |
| fosB | neg | 0,55 | 0,53 | 0,15 | 0,28 | metallothiol transferase |
| | POS | 0,43 | 0,47 | 0,85 | 0,72 | |
| qacA | neg | 1,00 | 1,00 | 1,00 | 0,98 | quaternary ammonium compound resistance protein A |
| | POS | 0,00 | 0,00 | 0,00 | 0,02 | |
| qacC (total) | neg | 1,00 | 1,00 | 1,00 | 1,00 | quaternary ammonium compound resistance protein C |
| qacC (consensus) | neg | 1,00 | 1,00 | 1,00 | 1,00 | |

| | | | | | | |
|---|---|---|---|---|---|---|
| qacC (equine) | neg | 1,00 | 1,00 | 1,00 | 1,00 | |
| qacC (SA5) | neg | 1,00 | 1,00 | 1,00 | 1,00 | |
| qacC (Ssap) | neg | 1,00 | 1,00 | 1,00 | 1,00 | |
| qacC (ST94) | neg | 1,00 | 1,00 | 1,00 | 1,00 | |
| sdrM | POS | 1,00 | 1,00 | 1,00 | 1,00 | Transport-/Effluxprotein |
| vanA | neg | 1,00 | 1,00 | 1,00 | 1,00 | vancomycin resistance gene |
| vanB | neg | 1,00 | 0,96 | 1,00 | 1,00 | vancomycin resistance gene from enterococci and Clostridium |
| | POS | 0,00 | 0,04 | 0,00 | 0,00 | |
| vanZ | neg | 1,00 | 1,00 | 1,00 | 1,00 | teicoplanin resistance gene from enterococci |
| tst1 (consensus) | neg | 0,93 | 0,91 | 1,00 | 1,00 | toxic shock syndrome toxin 1 |
| | POS | 0,07 | 0,09 | 0,00 | 0,00 | |
| tst1 ("human" allele) | neg | 0,96 | 0,96 | 1,00 | 1,00 | |
| | POS | 0,02 | 0,02 | 0,00 | 0,00 | |
| tst1 ("bovine" allele) | neg | 0,98 | 0,96 | 1,00 | 1,00 | |
| | POS | 0,02 | 0,04 | 0,00 | 0,00 | |
| sea | neg | 0,93 | 0,89 | 0,79 | 0,70 | enterotoxin A |
| | POS | 0,07 | 0,11 | 0,21 | 0,30 | |
| sea (N315) / sep | neg | 1,00 | 1,00 | 1,00 | 1,00 | |
| seb | neg | 0,98 | 0,98 | 1,00 | 1,00 | enterotoxin B |
| | POS | 0,00 | 0,02 | 0,00 | 0,00 | |
| sec | neg | 0,93 | 0,91 | 1,00 | 1,00 | enterotoxin C |
| | POS | 0,07 | 0,09 | 0,00 | 0,00 | |
| sed | neg | 0,86 | 0,82 | 0,64 | 0,53 | enterotoxin D |
| | POS | 0,09 | 0,18 | 0,36 | 0,47 | |
| see | neg | 1,00 | 1,00 | 1,00 | 1,00 | enterotoxin E |
| seg | neg | 0,73 | 0,84 | 0,76 | 1,00 | enterotoxin G |
| | POS | 0,27 | 0,16 | 0,24 | 0,00 | |
| seh | neg | 0,96 | 0,98 | 1,00 | 0,91 | enterotoxin H |
| | POS | 0,05 | 0,02 | 0,00 | 0,09 | |
| sei | neg | 0,73 | 0,84 | 0,76 | 1,00 | enterotoxin I |
| | POS | 0,27 | 0,16 | 0,24 | 0,00 | |
| sej | neg | 0,91 | 0,84 | 0,64 | 0,53 | enterotoxin J |
| | POS | 0,09 | 0,16 | 0,36 | 0,47 | |
| sek | neg | 1,00 | 1,00 | 1,00 | 1,00 | enterotoxin K |
| sel | neg | 0,93 | 0,91 | 1,00 | 1,00 | enterotoxin L |
| | POS | 0,07 | 0,09 | 0,00 | 0,00 | |
| selm | neg | 0,73 | 0,84 | 0,76 | 1,00 | enterotoxin M |
| | POS | 0,27 | 0,16 | 0,24 | 0,00 | |
| seln (consensus) | neg | 0,73 | 0,84 | 0,76 | 1,00 | enterotoxin N |
| | POS | 0,27 | 0,16 | 0,24 | 0,00 | |
| seln (other than RF122) | neg | 0,73 | 0,84 | 0,76 | 1,00 | |
| | POS | 0,27 | 0,16 | 0,24 | 0,00 | |
| selo | neg | 0,73 | 0,84 | 0,76 | 1,00 | enterotoxin O |
| | POS | 0,27 | 0,16 | 0,24 | 0,00 | |
| egc (total) | neg | 0,73 | 0,84 | 0,76 | 1,00 | egc cluster |
| | POS | 0,27 | 0,16 | 0,24 | 0,00 | |
| seq | neg | 1,00 | 1,00 | 1,00 | 1,00 | enterotoxin Q |
| ser | neg | 0,91 | 0,84 | 0,64 | 0,53 | enterotoxin R |
| | POS | 0,09 | 0,16 | 0,36 | 0,47 | |
| selu | neg | 0,73 | 0,84 | 0,76 | 1,00 | enterotoxin U and/or Y |

| | | | | | | |
|---|---|---|---|---|---|---|
| | POS | 0,27 | 0,16 | 0,24 | 0,00 | |
| ORF CM14 | neg | 0,89 | 0,80 | 0,85 | 1,00 | enterotoxin-like protein ORF CM14 |
| | POS | 0,11 | 0,20 | 0,15 | 0,00 | |
| lukF | POS | 1,00 | 1,00 | 1,00 | 1,00 | haemolysin gamma /leukocidin, component B |
| lukS | POS | 1,00 | 1,00 | 1,00 | 1,00 | haemolysin gamma /leukocidin, component C |
| hlgA | POS | 1,00 | 1,00 | 1,00 | 1,00 | haemolysin gamma, component A |
| lukF-PV | neg | 1,00 | 1,00 | 1,00 | 1,00 | Panton Valentine leukocidin, F component |
| lukS-PV | neg | 1,00 | 1,00 | 1,00 | 1,00 | Panton Valentine leukocidin, S component |
| lukF-PV (P83) | neg | 0,73 | 0,78 | 0,91 | 1,00 | F component from hypothetical leukocidin from ruminants |
| | POS | 0,27 | 0,22 | 0,09 | 0,00 | |
| lukM | neg | 0,75 | 0,78 | 0,91 | 1,00 | S component from hypothetical leukocidin from ruminants |
| | POS | 0,25 | 0,22 | 0,09 | 0,00 | |
| lukD | neg | 0,43 | 0,09 | 0,03 | 0,09 | leukocidin, D component |
| | POS | 0,57 | 0,91 | 0,97 | 0,92 | |
| lukE | neg | 0,43 | 0,13 | 0,00 | 0,09 | leukocidin, E component |
| | POS | 0,57 | 0,87 | 1,00 | 0,92 | |
| lukX | POS | 1,00 | 1,00 | 1,00 | 1,00 | leukocidin/haemolysin toxin family protein |
| lukY | POS | 1,00 | 1,00 | 1,00 | 1,00 | leukocidin/haemolysin toxin family protein |
| lukY (ST30+ST45) | neg | 1,00 | 1,00 | 1,00 | 1,00 | |
| hl | POS | 1,00 | 1,00 | 1,00 | 1,00 | putative membrane protein |
| hla | neg | 0,00 | 0,00 | 0,00 | 0,06 | haemolysin alpha |
| | POS | 1,00 | 1,00 | 1,00 | 0,94 | |
| hlIII (consensus) | POS | 1,00 | 1,00 | 1,00 | 1,00 | putative membrane protein |
| hlIII (other than RF122) | neg | 0,25 | 0,20 | 0,09 | 0,00 | |
| | POS | 0,75 | 0,80 | 0,91 | 1,00 | |
| hlb probe 1, 2 | neg | 0,36 | 0,11 | 0,00 | 0,09 | haemolysin beta |
| | POS | 0,61 | 0,89 | 1,00 | 0,92 | |
| hlb probe 3 | neg | 0,41 | 0,09 | 0,09 | 0,09 | |
| | POS | 0,59 | 0,91 | 0,91 | 0,92 | |
| un-disrupted hlb | neg | 0,14 | 0,09 | 0,09 | 0,26 | |
| | POS | 0,86 | 0,91 | 0,91 | 0,75 | |
| sak | neg | 0,93 | 0,89 | 0,76 | 0,68 | staphylokinase |
| | POS | 0,07 | 0,11 | 0,24 | 0,32 | |
| chp | neg | 1,00 | 1,00 | 0,97 | 1,00 | chemotaxis inhibiting protein |
| | POS | 0,00 | 0,00 | 0,03 | 0,00 | |
| scn | neg | 0,93 | 0,89 | 0,76 | 0,68 | staphylococcal complement inhibitor |
| | POS | 0,07 | 0,11 | 0,24 | 0,32 | |
| etA | neg | 1,00 | 1,00 | 1,00 | 1,00 | exfoliative toxin A |
| etB | neg | 1,00 | 1,00 | 1,00 | 1,00 | exfoliative toxin B |
| etD | neg | 1,00 | 1,00 | 1,00 | 1,00 | exfoliative toxin D |
| edinA | neg | 1,00 | 1,00 | 1,00 | 1,00 | epidermal cell differentiation inhibitor A |
| edinB | neg | 1,00 | 1,00 | 1,00 | 1,00 | epidermal cell differentiation inhibitor B |
| edinC | neg | 1,00 | 1,00 | 1,00 | 1,00 | epidermal cell differentiation inhibitor C |
| ACME (total) | neg | 1,00 | 1,00 | 1,00 | 1,00 | ACME locus |

| | | | | | | |
|---|---|---|---|---|---|---|
| arcA-SCC | neg | 1,00 | 1,00 | 1,00 | 1,00 | ACME locus |
| arcB-SCC | neg | 1,00 | 1,00 | 1,00 | 1,00 | ACME locus: ornithincarbamoyltransferase |
| arcC-SCC | neg | 1,00 | 1,00 | 1,00 | 1,00 | ACME locus: carbamatkinase |
| arcD-SCC | neg | 1,00 | 1,00 | 1,00 | 1,00 | ACME locus: arginine/ornithine antiporter |
| aur (consensus) | POS | 1,00 | 1,00 | 1,00 | 1,00 | aureolysin |
| aur (other than MRSA252) | neg | 0,36 | 0,09 | 0,00 | 0,09 | |
| | POS | 0,64 | 0,91 | 1,00 | 0,92 | |
| aur (MRSA252) | neg | 0,64 | 0,91 | 1,00 | 0,92 | |
| | POS | 0,36 | 0,09 | 0,00 | 0,09 | |
| splA | neg | 0,39 | 0,09 | 0,00 | 0,09 | serin-protease A |
| | POS | 0,61 | 0,91 | 1,00 | 0,92 | |
| splB | neg | 0,36 | 0,09 | 0,00 | 0,09 | serin-protease B |
| | POS | 0,64 | 0,91 | 1,00 | 0,92 | |
| splE | neg | 0,75 | 0,36 | 0,27 | 0,21 | serin-protease E |
| | POS | 0,25 | 0,64 | 0,73 | 0,79 | |
| sspA | POS | 1,00 | 1,00 | 1,00 | 1,00 | glutamylendopeptidase |
| sspB | POS | 1,00 | 1,00 | 1,00 | 1,00 | staphopain B, protease |
| sspP (consensus) | POS | 1,00 | 1,00 | 1,00 | 1,00 | staphopain A, protease |
| sspP (other than ST93) | POS | 1,00 | 1,00 | 1,00 | 1,00 | |
| setC | POS | 1,00 | 1,00 | 1,00 | 1,00 | staphylococcal exotoxin-like protein |
| ssl01/set6 probe1 11 | neg | 0,48 | 0,44 | 0,46 | 0,28 | staphylococcal superantigen-like protein 1 |
| | POS | 0,52 | 0,56 | 0,55 | 0,72 | |
| ssl01/set6 probe2 11 | neg | 0,73 | 0,89 | 0,79 | 0,83 | |
| | POS | 0,27 | 0,11 | 0,21 | 0,17 | |
| ssl01/set6 probe2 12 | neg | 0,64 | 0,82 | 0,85 | 0,89 | |
| | POS | 0,36 | 0,18 | 0,15 | 0,11 | |
| ssl01/set6 probe4 11 | neg | 0,43 | 0,38 | 0,18 | 0,21 | |
| | POS | 0,55 | 0,60 | 0,82 | 0,79 | |
| ssl01/set6 probeRF122 | neg | 0,66 | 0,62 | 0,82 | 0,89 | |
| | POS | 0,25 | 0,36 | 0,18 | 0,11 | |
| ssl01/set6 (COL) | neg | 0,84 | 0,53 | 0,58 | 0,38 | |
| | POS | 0,16 | 0,47 | 0,42 | 0,62 | |
| ssl01/set6 (MW2+MSSA476) | neg | 0,80 | 0,93 | 1,00 | 0,89 | |
| | POS | 0,21 | 0,07 | 0,00 | 0,11 | |
| ssl01/set6 (MRSA252) | neg | 0,98 | 0,96 | 0,88 | 0,94 | |
| | POS | 0,02 | 0,04 | 0,12 | 0,06 | |
| ssl01/set6 (RF122) | neg | 0,82 | 0,76 | 0,88 | 0,89 | |
| | POS | 0,18 | 0,24 | 0,12 | 0,11 | |
| ssl01/set6 (other alleles) | neg | 0,91 | 0,91 | 0,76 | 1,00 | |
| | POS | 0,09 | 0,09 | 0,24 | 0,00 | |
| ssl02/set7 | neg | 0,23 | 0,09 | 0,00 | 0,09 | staphylococcal superantigen-like protein 2 |
| | POS | 0,71 | 0,91 | 1,00 | 0,92 | |
| ssl03/set8 probe 1 | neg | 0,41 | 0,13 | 0,00 | 0,09 | staphylococcal superantigen-like protein 3 |
| | POS | 0,57 | 0,87 | 1,00 | 0,92 | |
| ssl03/set8 (MRSA252, SAR0424) | neg | 1,00 | 1,00 | 1,00 | 1,00 | |
| ssl04/set9 | neg | 0,43 | 0,20 | 0,09 | 0,09 | |

| | | | | | | |
|---|---|---|---|---|---|---|
| | POS | 0,55 | 0,80 | 0,88 | 0,92 | |
| ssl04/set9 | neg | 0,59 | 0,91 | 1,00 | 0,89 | staphylococcal superantigen-like protein 4 |
| (MRSA252, SAR0425) | POS | 0,27 | 0,09 | 0,00 | 0,09 | |
| ssl05/set3 | neg | 0,68 | 0,29 | 0,12 | 0,11 | staphylococcal superantigen-like protein 5 |
| probe 1 | POS | 0,32 | 0,71 | 0,88 | 0,89 | |
| ssl05/set3 | neg | 0,61 | 0,91 | 1,00 | 0,92 | |
| (MRSA252) | POS | 0,39 | 0,09 | 0,00 | 0,09 | |
| ssl06/set21 | neg | 0,73 | 0,47 | 0,55 | 0,28 | staphylococcal superantigen-like protein 6 |
| | POS | 0,27 | 0,53 | 0,46 | 0,72 | |
| ssl08/set12 probe 1, 2 | neg | 0,43 | 0,16 | 0,00 | 0,09 | staphylococcal superantigen-like protein 8 |
| | POS | 0,57 | 0,84 | 1,00 | 0,92 | |
| ssl09/set5 | neg | 0,43 | 0,13 | 0,15 | 0,11 | staphylococcal superantigen-like protein 9 |
| probe 1, 2 | POS | 0,57 | 0,87 | 0,85 | 0,89 | |
| ssl09/set5 | neg | 0,64 | 0,91 | 1,00 | 0,92 | |
| (MRSA252) | POS | 0,36 | 0,09 | 0,00 | 0,09 | |
| ssl11/set2 | neg | 0,91 | 0,53 | 0,58 | 0,36 | staphylococcal superantigen-like protein 11 |
| (COL) | POS | 0,09 | 0,47 | 0,42 | 0,64 | |
| ssl11+set2(M | neg | 1,00 | 1,00 | 0,88 | 1,00 | |
| u50+N315) | POS | 0,00 | 0,00 | 0,12 | 0,00 | |
| ssl11+set2(M | neg | 0,86 | 0,87 | 0,91 | 0,92 | |
| W2+MSSA476) | POS | 0,14 | 0,13 | 0,09 | 0,09 | |
| ssl11/set2 | neg | 0,64 | 0,91 | 1,00 | 0,92 | |
| (MRSA252) | POS | 0,36 | 0,09 | 0,00 | 0,09 | |
| setB1 | POS | 1,00 | 1,00 | 1,00 | 1,00 | staphylococcal exotoxin-like protein, 2nd locus |
| setB2 | neg | 0,39 | 0,09 | 0,00 | 0,13 | |
| | POS | 0,61 | 0,91 | 1,00 | 0,87 | |
| setB2 | neg | 0,64 | 0,91 | 1,00 | 0,92 | |
| (MRSA252) | POS | 0,36 | 0,09 | 0,00 | 0,09 | |
| setB3 | neg | 0,36 | 0,09 | 0,00 | 0,09 | |
| | POS | 0,64 | 0,91 | 1,00 | 0,92 | |
| setB3 | neg | 0,64 | 0,91 | 1,00 | 0,92 | |
| (MRSA252) | POS | 0,36 | 0,09 | 0,00 | 0,09 | |
| cap 1 (total) | neg | 0,96 | 1,00 | 1,00 | 1,00 | capsule type 1 |
| | POS | 0,05 | 0,00 | 0,00 | 0,00 | |
| capH1 | neg | 0,96 | 1,00 | 1,00 | 1,00 | capsular polysaccharide synthesis enzyme |
| | POS | 0,05 | 0,00 | 0,00 | 0,00 | |
| capJ1 | neg | 0,93 | 1,00 | 1,00 | 1,00 | O antigen polymerase |
| | POS | 0,07 | 0,00 | 0,00 | 0,00 | |
| capK1 | neg | 1,00 | 1,00 | 1,00 | 1,00 | capsular polysaccharide biosynhesis protein |
| cap 5 (total) | neg | 0,30 | 0,31 | 0,15 | 0,11 | capsule type 5 |
| | POS | 0,71 | 0,69 | 0,85 | 0,89 | |
| capH5 | neg | 0,30 | 0,31 | 0,15 | 0,11 | capsular polysaccharide synthesis enzyme |
| | POS | 0,71 | 0,69 | 0,85 | 0,89 | |
| capJ5 | neg | 0,30 | 0,31 | 0,15 | 0,11 | O antigen polymerase |
| | POS | 0,71 | 0,69 | 0,85 | 0,89 | |
| capK5 | neg | 0,30 | 0,31 | 0,15 | 0,11 | capsular polysaccharide biosynhesis protein |
| | POS | 0,71 | 0,69 | 0,85 | 0,89 | |
| cap 8 (total) | neg | 0,71 | 0,69 | 0,85 | 0,89 | capsule type 8 |
| | POS | 0,30 | 0,31 | 0,15 | 0,11 | |
| capH8 | neg | 0,71 | 0,69 | 0,85 | 0,89 | capsular polysaccharide synthesis enzyme |
| | POS | 0,30 | 0,31 | 0,15 | 0,11 | |
| capI8 | neg | 0,71 | 0,69 | 0,85 | 0,89 | capsular polysaccharide biosynhesis protein |
| | POS | 0,30 | 0,31 | 0,15 | 0,11 | |
| capJ8 | neg | 0,71 | 0,69 | 0,85 | 0,89 | O antigen polymerase |

| | | | | | | |
|---|---|---|---|---|---|---|
| capK8 | POS | 0,30 | 0,31 | 0,15 | 0,11 | capsular polysaccharide biosynhesis protein |
| | neg | 0,71 | 0,69 | 0,85 | 0,89 | |
| | POS | 0,30 | 0,31 | 0,15 | 0,11 | |
| icaA | neg | 0,02 | 0,07 | 0,03 | 0,00 | intercellular adhesion protein A |
| | POS | 0,98 | 0,93 | 0,97 | 1,00 | |
| icaC | neg | 0,02 | 0,09 | 0,06 | 0,00 | intercellular adhesion protein C |
| | POS | 0,98 | 0,91 | 0,94 | 1,00 | |
| icaD | neg | 0,02 | 0,09 | 0,06 | 0,00 | intercellular adhesion protein D |
| | POS | 0,98 | 0,91 | 0,94 | 1,00 | |
| bap | neg | 0,96 | 1,00 | 0,88 | 0,96 | surface protein involved in biofilm formation |
| | POS | 0,05 | 0,00 | 0,12 | 0,04 | |
| bbp (total) | neg | 0,09 | 0,20 | 0,15 | 0,21 | bone sialoprotein-binding protein |
| | POS | 0,91 | 0,80 | 0,85 | 0,79 | |
| bbp (consensus) | neg | 0,11 | 0,22 | 0,21 | 0,23 | |
| | POS | 0,89 | 0,78 | 0,79 | 0,77 | |
| bbp (MRSA252) | neg | 1,00 | 1,00 | 1,00 | 1,00 | |
| bbp (RF122) | neg | 0,89 | 0,89 | 0,91 | 1,00 | |
| | POS | 0,09 | 0,11 | 0,09 | 0,00 | |
| clfA (total) | POS | 1,00 | 1,00 | 1,00 | 1,00 | clumping factor A |
| clfA (consensus) | POS | 1,00 | 1,00 | 1,00 | 1,00 | |
| clfB (total) | POS | 1,00 | 1,00 | 1,00 | 1,00 | clumping factor B |
| clfB (consensus) | POS | 1,00 | 1,00 | 1,00 | 1,00 | |
| clfB (COL+Mu50) | neg | 0,86 | 0,53 | 0,46 | 0,36 | |
| | POS | 0,09 | 0,47 | 0,55 | 0,64 | |
| clfB (MW2) | neg | 0,55 | 0,89 | 0,79 | 0,81 | |
| | POS | 0,46 | 0,11 | 0,21 | 0,19 | |
| cna | neg | 0,52 | 0,71 | 0,94 | 0,83 | collagen-binding adhesin |
| | POS | 0,48 | 0,29 | 0,06 | 0,17 | |
| ebh (consensus) | neg | 0,00 | 0,00 | 0,15 | 0,02 | cell wall associated fibronectin-binding protein |
| | POS | 1,00 | 1,00 | 0,85 | 0,98 | |
| ebpS (total) | POS | 1,00 | 1,00 | 1,00 | 1,00 | cell surface elastin-binding protein |
| ebpS probe 612 | neg | 0,43 | 0,09 | 0,00 | 0,09 | |
| | POS | 0,57 | 0,91 | 1,00 | 0,92 | |
| ebpS probe 614 | POS | 1,00 | 1,00 | 1,00 | 1,00 | |
| ebpS (01-1111) | neg | 0,57 | 0,91 | 1,00 | 0,92 | |
| | POS | 0,43 | 0,09 | 0,00 | 0,09 | |
| ebpS (COL) | neg | 0,43 | 0,44 | 0,27 | 0,21 | |
| | POS | 0,57 | 0,56 | 0,73 | 0,79 | |
| eno | POS | 1,00 | 1,00 | 1,00 | 1,00 | enolase |
| fib | neg | 0,36 | 0,09 | 0,00 | 0,09 | fibrinogen-binding protein |
| | POS | 0,64 | 0,91 | 1,00 | 0,92 | |
| fib (MRSA252) | neg | 0,64 | 0,91 | 1,00 | 0,92 | |
| | POS | 0,36 | 0,09 | 0,00 | 0,09 | |
| fnbA (total) | neg | 0,00 | 0,02 | 0,00 | 0,00 | fibronectin-binding protein A |
| | POS | 1,00 | 0,98 | 1,00 | 1,00 | |
| fnbA (consensus) | neg | 0,00 | 0,02 | 0,00 | 0,00 | |
| | POS | 1,00 | 0,98 | 1,00 | 1,00 | |
| fnbA (COL) | neg | 0,75 | 0,51 | 0,30 | 0,30 | |
| | POS | 0,25 | 0,49 | 0,70 | 0,70 | |
| fnbA (MRSA252) | neg | 0,93 | 1,00 | 0,97 | 0,98 | |
| | POS | 0,07 | 0,00 | 0,03 | 0,02 | |

| | | | | | | |
|---|---|---|---|---|---|---|
| fnbA (Mu50+MW2) | neg | 0,96 | 0,98 | 0,88 | 0,92 | |
| | POS | 0,05 | 0,02 | 0,12 | 0,09 | |
| fnbA (RF122) | neg | 0,91 | 0,89 | 0,91 | 1,00 | |
| | POS | 0,09 | 0,11 | 0,09 | 0,00 | |
| fnbB (total) | neg | 0,18 | 0,18 | 0,30 | 0,06 | fibronectin-binding protein B |
| | POS | 0,82 | 0,82 | 0,70 | 0,94 | |
| fnbB (COL) | neg | 0,96 | 0,53 | 0,58 | 0,38 | |
| | POS | 0,05 | 0,47 | 0,42 | 0,62 | |
| fnbB (MW2) | neg | 0,93 | 0,96 | 0,94 | 0,89 | |
| | POS | 0,07 | 0,04 | 0,06 | 0,11 | |
| fnbB (ST15) | neg | 0,93 | 1,00 | 0,97 | 1,00 | |
| | POS | 0,07 | 0,00 | 0,03 | 0,00 | |
| fnbB (ST45-2) | neg | 1,00 | 1,00 | 1,00 | 1,00 | |
| sasG (total) | neg | 0,48 | 0,20 | 0,36 | 0,17 | Staphylococcus aureus surface protein G |
| | POS | 0,52 | 0,80 | 0,64 | 0,83 | |
| sasG (COL+Mu50) | neg | 0,68 | 0,27 | 0,36 | 0,26 | |
| | POS | 0,32 | 0,73 | 0,64 | 0,75 | |
| sasG (other than MRSA252+RF122) | neg | 0,59 | 0,20 | 0,36 | 0,17 | |
| | POS | 0,41 | 0,80 | 0,64 | 0,83 | |
| sdrC (total) | POS | 1,00 | 1,00 | 1,00 | 1,00 | serine-aspartate repeat protein C |
| sdrC (consensus) | POS | 1,00 | 1,00 | 1,00 | 1,00 | |
| sdrC (COL) | neg | 0,77 | 0,44 | 0,58 | 0,36 | |
| | POS | 0,23 | 0,56 | 0,42 | 0,64 | |
| sdrC (Mu50) | neg | 0,43 | 0,71 | 0,52 | 0,72 | |
| | POS | 0,57 | 0,29 | 0,49 | 0,28 | |
| sdrC (other than MRSA252+RF122) | neg | 0,61 | 0,29 | 0,09 | 0,09 | |
| | POS | 0,39 | 0,71 | 0,91 | 0,92 | |
| sdrD (total) | neg | 0,23 | 0,27 | 0,39 | 0,23 | serine-aspartate repeat protein D |
| | POS | 0,77 | 0,73 | 0,61 | 0,77 | |
| sdrD (consensus) | neg | 0,23 | 0,27 | 0,39 | 0,23 | |
| | POS | 0,77 | 0,73 | 0,61 | 0,77 | |
| sdrD (COL+MW2) | neg | 0,86 | 0,56 | 0,61 | 0,43 | |
| | POS | 0,14 | 0,44 | 0,39 | 0,57 | |
| sdrD (other) | neg | 0,93 | 1,00 | 0,97 | 1,00 | |
| | POS | 0,07 | 0,00 | 0,03 | 0,00 | |
| vwb (total) | POS | 1,00 | 1,00 | 1,00 | 1,00 | van Willebrand factor- binding protein |
| vwb (consensus) | POS | 1,00 | 1,00 | 1,00 | 1,00 | |
| vwb (COL+MW2) | neg | 0,84 | 0,51 | 0,58 | 0,28 | |
| | POS | 0,16 | 0,49 | 0,42 | 0,72 | |
| vwb (MRSA252) | neg | 1,00 | 1,00 | 1,00 | 1,00 | |
| vwb (Mu50) | neg | 0,93 | 1,00 | 0,85 | 1,00 | |
| | POS | 0,07 | 0,00 | 0,15 | 0,00 | |
| vwb (RF122) | neg | 0,82 | 0,67 | 0,85 | 0,89 | |
| | POS | 0,18 | 0,33 | 0,15 | 0,11 | |
| mprF (COL+MW2) | neg | 0,57 | 0,13 | 0,03 | 0,11 | defensin resistance protein |
| | POS | 0,43 | 0,87 | 0,82 | 0,89 | |

| | | | | | | |
|---|---|---|---|---|---|---|
| isdA (consensus) | POS | 1,00 | 1,00 | 1,00 | 1,00 | transferrin-binding protein |
| lmrP (other than RF122)_probe2 | neg | 0,25 | 0,29 | 0,15 | 0,00 | hypothetical protein, similar to integral membrane protein LmrP |
| | POS | 0,75 | 0,71 | 0,85 | 1,00 | |
| lmrP (RF122)_probe2 | neg | 0,75 | 0,71 | 0,85 | 1,00 | |
| | POS | 0,25 | 0,29 | 0,15 | 0,00 | |
| hsdS1 (RF122) | neg | 0,91 | 0,89 | 0,91 | 1,00 | type I site-specific deoxyribonuclease subunit, 1st locus |
| | POS | 0,09 | 0,11 | 0,09 | 0,00 | |
| hsdS2 (Mu50+N315+COL+USA300+NCTC8325) | neg | 0,84 | 0,49 | 0,46 | 0,34 | type I site-specific deoxyribonuclease subunit, 2nd locus |
| | POS | 0,16 | 0,51 | 0,55 | 0,66 | |
| hsdS2 (RF122) | neg | 0,91 | 0,89 | 0,91 | 1,00 | |
| | POS | 0,09 | 0,11 | 0,09 | 0,00 | |
| hsdS2 (MRSA252) | neg | 1,00 | 1,00 | 1,00 | 1,00 | |
| hsdS3 (all other than RF122+ MRSA252) | neg | 0,66 | 0,38 | 0,30 | 0,28 | type I site-specific deoxyribonuclease subunit, 3rd locus |
| | POS | 0,27 | 0,62 | 0,70 | 0,72 | |
| hsdS3 (COL+USA300+NCTC8325+MW2+MSSA476+RF122) | neg | 0,71 | 0,36 | 0,49 | 0,28 | |
| | POS | 0,30 | 0,64 | 0,52 | 0,72 | |
| hsdS3 (Mu50+N315) | neg | 0,96 | 1,00 | 0,61 | 0,94 | |
| | POS | 0,05 | 0,00 | 0,39 | 0,06 | |
| hsdS3 (MRSA252) | neg | 1,00 | 1,00 | 1,00 | 1,00 | |
| hsdSx (etd) | neg | 1,00 | 0,96 | 1,00 | 0,98 | type I site-specific deoxyribonuclease subunit, unknown locus |
| | POS | 0,00 | 0,04 | 0,00 | 0,02 | |
| Q2FXC0 | neg | 0,86 | 0,51 | 0,58 | 0,26 | hypothetical protein, located next to serine protease operon |
| | POS | 0,14 | 0,49 | 0,42 | 0,75 | |
| Q7A4X2 | neg | 0,57 | 0,58 | 0,70 | 0,89 | hypothetical protein |
| | POS | 0,43 | 0,42 | 0,30 | 0,11 | |
| hysA1 (MRSA252) | neg | 1,00 | 0,96 | 1,00 | 0,98 | hyaluronate lyase, 1st/2nd locus |
| | POS | 0,00 | 0,04 | 0,00 | 0,02 | |
| hysA1 (MRSA252+ RF122) and/or hysA2 (consensus) | POS | 1,00 | 1,00 | 1,00 | 1,00 | |
| hysA1 (MRSA252+ RF122) and/or hysA2 (COL+USA300) | neg | 0,43 | 0,40 | 0,49 | 0,26 | |
| | POS | 0,57 | 0,60 | 0,52 | 0,75 | |

| | | | | | | |
|---|---|---|---|---|---|---|
| hysA2 (all other than MRSA252) | neg | 0,73 | 0,40 | 0,36 | 0,28 | hyaluronate lyase, 2nd locus |
| | POS | 0,27 | 0,60 | 0,64 | 0,72 | |
| hysA2 (COL+USA300+NCTC8325) | neg | 0,43 | 0,33 | 0,49 | 0,26 | |
| | POS | 0,57 | 0,67 | 0,52 | 0,75 | |
| hysA2 (all other than COL+USA300+NCTC8325) | neg | 0,46 | 0,56 | 0,42 | 0,72 | |
| | POS | 0,55 | 0,44 | 0,58 | 0,28 | |
| hysA2 (all other than COL+USA300+NCTC8325) | neg | 0,46 | 0,56 | 0,42 | 0,72 | |
| | POS | 0,55 | 0,44 | 0,58 | 0,28 | |
